# Factors Associated with Postpartum Post-Traumatic Stress Disorder (PTSD) Following Obstetric Violence: A Cross-Sectional Study

**DOI:** 10.3390/jpm11050338

**Published:** 2021-04-24

**Authors:** Sergio Martinez-Vázquez, Julián Rodríguez-Almagro, Antonio Hernández-Martínez, Juan Miguel Martínez-Galiano

**Affiliations:** 1 Department of Nursing, University of Jaen, 23071 Jaen, Spain; svazquez@ujaen.es (S.M.-V.); jgaliano@ujaen.es (J.M.M.-G.); 2 Department of Nursing, Faculty of Nursing of Ciudad Real, The University of Castilla-La Mancha, 13071 Ciudad Real, Spain; JulianJ.Rodriguez@uclm.es; 3 Consortium for Biomedical Research in Epidemiology and Public Health (CIBERESP), 28029 Madrid, Spain

**Keywords:** obstetric violence, post-traumatic stress disorder (PTSD), associated factors, puerperium, postpartum

## Abstract

To determine the association between experiencing obstetric violence and the incidence of postpartum post-traumatic stress disorder (PTSD). A cross-sectional study with puerperal women was conducted in Spain following ethical approval. The Perinatal Posttraumatic Stress Disorder Questionnaire (PPQ) was administered online. Sociodemographic, clinical, and obstetric violence variables and the risk of dichotomized PTSD (low/high) were studied by bivariate and multivariate analysis with binary logistic regression. 955 women were invited to participate. 53 women refused to participate, three did not complete all survey questions and, finally, 899 women were included. The risk of PTSD (score ≥ 19) using the PPQ was 12.7% (114). The mean score was 9.10 points (SD = 8.52). Risk factors identified were having a delivery plan that was not respected (aOR: 2.85, 95% CI 1.56–5.21), elective caesarean delivery (aOR: 2.53, 95% CI 1.02–2.26), emergency caesarean section (aOR: 3.58, 95% CI 1.83–6.99), admission of the newborn to the neonatal intermediate care unit (aOR: 4.95, 95% CI 2.36–10.36), admission to the intensive care unit (aOR: 2.25, 95% CI 1.02–4.97), formula feeding on discharge (aOR: 3.57, 95% CI 1.32–9.62), verbal obstetric violence (aOR: 5.07, 95% CI 2.98–8.63), and psycho-affective obstetric violence (aOR: 2.61, 95% CI 1.45–4.67). Various clinical practices were identified with the risk of PTSD, highlighting various types of obstetric violence. Partner support and early breastfeeding were identified as protective factors. Sensitizing professionals is essential to prevent the risk of PTSD.

## 1. Introduction

Pregnancy, childbirth, and postpartum are periods in which a woman’s risk of developing a mental disorder increases; despite this, mental health is an aspect that does not usually receive a lot of attention in the care provided during these periods [1,2].

Postpartum post-traumatic stress disorder (PTSD) is one of these disorders and may be present in 0.8–43% of women, depending on whether the assessment uses only self-declared symptoms, the diagnostic criteria of the DSM, or if the study has been carried out at a community level or in the at-risk population [3,4,5,6,7,8].

Women who experience this disorder report re-experimentation of the event, a feeling of disconnection from the baby, absence of reality, nightmares, irritability, rejection of new motherhood, or may even develop tocophobia (the fear of pregnancy and childbirth) [9,10,11,12,13,14].

The maximum expression of these symptoms appears between 4–6 weeks postpartum, although the symptoms can remain for months or years later, and even in future pregnancies [3,15].

Different variables have been associated with the risk of developing PTSD, such as having been abused during childhood [16], exposure to trauma [17], the type of delivery, the Kristeller maneuver being performed in the expulsive period, having third or fourth-degree perineal tears [18], having a postpartum hemorrhage [19], or being afraid of childbirth [20]. In addition, age, parity, and having resources, such as coping skills, have also been associated with the incidence of PTSD [21,22].

Postpartum PTSD affects maternal morbidity [11,14] It can also affect the couple, the familial environment, the family, and, especially, the baby [12,23,24] It has also been associated with a lower rate of breastfeeding initiation [24] and a higher incidence of low birth weight [4].

In the care that women receive during childbirth, they may experience obstetric violence, also called incorrect or inappropriate treatment [25], either physically or emotionally with inadequate clinical care or with violation of the principle of autonomy [26]. In some countries, a frequency of 35.4% has been reported for physical, verbal, or discrimination abuse [27], reaching 91.7% in some studies for abuse and lack of respect [28]. In other countries such as Spain, the prevalence of obstetric violence is around 67% [29]. Due to this, the World Health Organization (WHO) has carried out a strategy aimed at health professionals to alert them on this issue [30].

Obstetric violence and the incidence of PSTD have been studied by several authors [31,32]. In a meta-analysis with 50 studies, it was suggested that a negative experience during delivery was a risk factor for the appearance of PTSD, prompted, among other reasons, by lack of support or type of delivery, i.e., whether it was instrumental or caesarean [31]. In addition, subjective distress generated by experiences during childbirth appears to be an important predictor for the development of PTSD and was the main risk factor in the 31 studies analyzed in a systematic review carried out in 2012 [32].

Establishing a possible association between experiencing obstetric violence and the incidence of PTSD will help sensitize health professionals who provide care during pregnancy, childbirth, and the postpartum period; promoting strategies with correct and evidence-based clinical practices and reducing the medicalization of the natural childbirth process [33]. In this way, the incidence of PTSD and its resulting consequences for maternal and newborn health could be reduced [11,12,34]. Therefore, the present study intended to determine if there is an association between experiencing obstetric violence and the risk of PTSD.

## 2. Methodology

### 2.1. Design and Participants

A cross-sectional study was conducted with women who had given birth in Spain. The inclusion criteria were postpartum women whose delivery had been in the last 12 months, and who were of legal age. The only exclusion criteria were not being able to understand the Spanish language, and that a minimum of one month had not elapsed since the date of birth. Data collection was carried out from September to December 2019. This study received the approval of the Research Ethics Committee of the province of Jaen with reference number TD-VCDEPP-2019/1417-N-19. All women were required to read the study information and then give their digital consent by checking a box, including the fact that participation was entirely voluntary with anonymity guaranteed.

For calculating the sample size, the maximum modelling criterion was used where 10 events (women at risk of PTSD) are included for each independent variable to be introduced in the multivariate model [35]. Considering the prevalence of PTSD risk was 10% in previous studies in Spain [36], 80 women at risk of PTSD would be needed to include eight variables and a minimum of 800 women included in the study.

### 2.2. Data Collection and Information Sources

Data was collected using a previously piloted online questionnaire that we had constructed for this purpose. The questionnaire collected information on sociodemographic variables, clinical variables, and variables related to the woman’s experience with childbirth care, among other information.

The main independent variable was obstetric violence and its three components, verbal, physical, and psycho-affective. A questionnaire was used to determine its occurrence by asking about various practices and situations that the woman had experienced during her labor process. (Annex 1. Obstetric violence questionnaire).

The main outcome variable was the risk of PTSD, which was assessed using the Perinatal Post-traumatic Stress Disorder Questionnaire (PPQ) [37]. The PPQ consists of 14 questions with Likert-type answers with scores ranging from 0 to 56 points. A high risk of PTSD score was considered as a score of 19 points or higher.

The dissemination of the questionnaire was carried out thanks to the collaboration of the different associations of Spanish midwives who distributed the questionnaire among the women to whom they provided their care.

### 2.3. Statistical Analysis

First, descriptive statistics were performed using absolute and relative frequencies for categorical variables and mean with standard deviation for quantitative variables. Next, a bivariate analysis was performed between the sociodemographic, clinical, and obstetric violence variables and the risk of PTSD (dichotomized as low risk/high risk). Crude (OR) and adjusted (aOR) odds ratios were estimated with corresponding 95% confidence intervals, utilizing a bivariate and multivariate analysis with binary logistic regression, respectively. For the multivariate analysis, the SPSS backward step procedure was used.

## 3. Results

### Characteristics of Participants

955 women were invited to participate. 53 women refused to participate, three did not complete all survey questions, and finally 899 women were included. The mean age of the participants was 35.2 years (SD = 4.25 years), and 87.9% (736) had their delivery within the network of Spanish public hospitals, 92.9% (835) had a planned pregnancy, and 96.6% (863) of the sample were Spanish. Further sociodemographic and clinical information that characterizes the sample can be found in Table 1.

**Figure 1:**
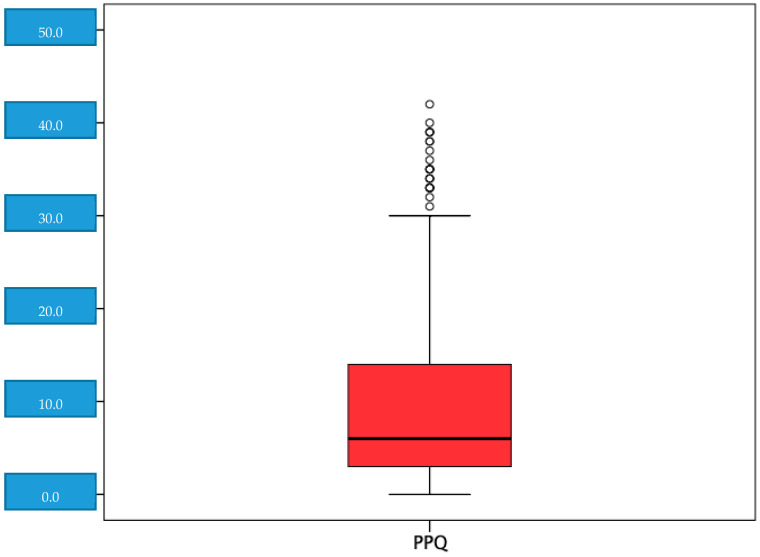
Distribution of the scores in the perinatal post-traumatic stress questionnaire (PPQ).

Subsequently, the risk of PTSD was determined (with a score ≥ of 19) and found 12.7% (114) had a high risk of PTSD, with a mean score in the PPQ questionnaire of 9.10 points (SD = 8.52) (Figure 1).

Next, a bivariate analysis was conducted, and an association between the risk of PTSD with 19 variables was observed, including global obstetric violence and the verbal, physical, and psycho-affective subtypes. (Table 2).

Finally, a multivariate analysis was performed where the following statistical associations were found about the risk of PTSD:

Risk factors were identified as having a delivery plan that was not respected (aOR: 2.85, 95% CI 1.56–5.21), have a scheduled caesarean delivery (aOR: 2.53, 95% CI 1.02–2.26), having an emergency caesarean section (aOR: 3.58, 95% CI 1.83–6.99), admission of the newborn to the neonatal intermediate care unit (aOR: 4.95, 95% CI 2.36–10.36), admission to the intensive care unit (aOR: 2.25, 95% CI 1.02–4.97), newborn formula-fed on discharge (aOR: 3.57, 95% CI 1.32–9.62), experiencing verbal obstetric violence (aOR: 5.07, 95% CI 2.98– 8.63) and psycho-affective obstetric violence (aOR: 2.61, 95% CI 1.45–4.67).

Protective factors were identified as initiating lactation in the first hour postpartum (aOR: 0.48, 95% CI 0.26–0.87), as well as the perception of support by the couple during pregnancy, childbirth and the puerperium, with an aOR of 0.16 (95% CI 0.04–0.63) for some support, an aOR of 0.17 (95% CI 0.05–0.57) for quite a bit of support, and an aOR of 0.17 (95% CI 0.06–0.53) for a lot of support.

## 4. Discussion

Approximately 13 out of 100 women had a high risk of PTSD. Women with a delivery plan that was not respected, had given birth by scheduled or urgent caesarean section, or had suffered verbal and psycho-affective violence had a higher incidence of PTSD. Mothers of infants admitted to intensive care, or whose infants were formula-fed at discharge also showed a higher incidence of this disorder. Those women who started breastfeeding in the first hour postpartum and felt supported by their partner had a lower risk of developing PTSD.

The study sample is representative of the reference population. The risk of PTSD was detected using a validated instrument [37] that has already been used previously in populations similar to ours [38]. Due to the nature of a questionnaire, there may be a selection bias associated with non-response; however, as the sample is large and representative, we do not think that the responses of those women who did not participate could differ too much from those who did form part of the sample. It is important to note that the number of women who did not respond was low, 53 in total (5.57%). The questions and possible answers were simple, understandable, and easy to understand for any education level, minimizing any possibility of information bias. The information was collected in a short time range, and although we cannot completely rule out a memory bias, we believe that its impact on the results is minimal. Completely ruling out a confounding bias is impossible, although attempts have been made to control this through study design and adjusting for confounding variables during data analysis. The absence of an official record where obstetric violence appears and the self-declared nature of the violence experienced by women is one of the limitations of the study as it is a subjective experience situation. The questionnaire was provided online, which may limit the participation of women who do not have access to the network. However, this would be a rare occurrence as the use of smartphones, tablets, or computers is common in the current population. The online questionnaire tool has been included in previous research as a method of data collection [18,39]. Dichotomizing Perinatal Post-traumatic Stress Disorder Questionnaire Score at 19 indicates that we believe there is little or no difference scoring between 1 and 18, or scoring between 19 and 56. The only difference occurs between those scores 18 and 19. The risk of PTSD was considered a score of ≥ 19 points. We dichotomized this variable at 19 because past research established this as the score at which risk for PTSD rises [37].

The PTSD incidence in our results is within the range found by Beck and Casavant [3] in their systematic review of 59 studies, where they placed the prevalence of PTSD between 0.8% and 26%. Our results are also very similar to those found by Vignato et al. [40], in a study developed in the United States, including four systematic reviews, two meta-analyses, and 11 more articles (not included in the previous ones), although the range reported was wide. Other authors found figures somewhat higher than those identified in our results. van Heumen et al. found that 17.4% of the participants met PTSD criteria, using data collected with a 35-item questionnaire that included a validated PTSD screening in a study conducted in the Netherlands, in which women with at least one traumatic experience during childbirth between 2005 and 2016 participated [22].

The appearance of PTSD was associated with a lack of respect and non-compliance with the birth plan by professionals, in line with what was reported in a cross-sectional study conducted in Spain with 2990 women [36] where they identified that a birth plan that professionals had respected was a protective factor against the development of PTSD. A caesarean delivery, whether scheduled or urgent, was associated with a higher incidence of PTSD, coinciding with that found by other authors [36]. Of the 5332 women who participated in a study conducted in England, 23% of women who underwent an emergency caesarean section and 16% of those who had a scheduled caesarean section reported at least 1 or more symptoms of PTSD, thus considering caesarean section a risk factor [41] Also consistent with our results, in a study conducted in Sweden, higher levels of mental distress were found in women who had an emergency or scheduled caesarean section [42]. These results, however, contrast with those found by Mahmoodi et al., in a study involving 240 women in Iran [43], where PTSD was not associated with the type of delivery the woman had experienced. Also in contrast with our findings are those of Olieman et al. [44], in their systematic review including three articles, which identified vaginal delivery as a risk factor, especially when the woman’s preference was to give birth by caesarean section, thus suggesting that a caesarean section may become a protective factor against PTSD.

The admission of the newborn to some type of unit, whether intermediate or intensive care, also showed an association with the appearance of PTSD. This contrasts with that reported in a study carried out by Aftyka et al. in Poland with 39 mothers whose babies had been admitted to the intensive care unit [45], in which no association between both variables was found. However, Kim et al. reported results similar to those found in our study [38], identifying an association between the appearance of early or late PTSD and neonates admitted to neonatal intensive care units. Furthermore, the PTSD screening method used in this study coincides with that used in our research, the Perinatal Post-traumatic Stress Disorder Questionnaire (PPQ). Other authors included not only the admission to intensive care units occurred but also the duration of admission. Lefkowitz et al. [46], found an association between a minimum stay of 30 days and the appearance of the disorder. This may be due to the mother-child pairing separation resulting from the admission, and this can cause stress to the mother.

Verbal and psycho-affective obstetric violence also show an association with the appearance of the PTSD, with verbal violence being the most likely to affect the development of this disorder. Appropriate verbal treatment, giving concrete and understandable information, as well as ensuring informed consent have been highlighted by van Dinter-Douma et al. [47], as elements that could help reduce fear during childbirth. The impact of a negative experience during childbirth and PTSD has been considered by the Spanish Ministry of Health in its national delivery care strategy, describing PTSD as an obstetric sequela [48].

Early breastfeeding, started in the baby’s first hour of life, was identified as a protective factor in the appearance of PTSD. These results coincide with Garthus-Niegel et al. [24], where they found an association between postpartum PTSD and not initiating breastfeeding. In this study, data was collected from the birth records of a Norwegian hospital and questionnaires administered to the 1480 women who participated. They also found an association between not maintaining this type of lactation during the first year and the appearance of PTSD. A systematic review that included 21 studies also found that postpartum PTSD was associated with lower breastfeeding rates, in line with our results [4]. Moreover, based on the results obtained, formula feeding at discharge emerged as a risk factor for PTSD. Imširagić et al. [49], in their study conducted in Croatia involving 259 women, identified lower levels of PTSD in those who continued exclusive breastfeeding for 6–9 weeks postpartum.

The perceived support received from the partner emerged as a protective factor, regardless of the degree of support. No studies exist to date that analyze the relationship between these two variables; highlighting the need for research to increase knowledge in this regard. Although other authors such as Van Heumen et al. [22] have studied support, but from the perspective of social support, identifying it as a protective factor for the appearance of the disorder. The perception of the woman who has support may make her feel protected and supported in the face of possible complications, inconveniences, or doubts that arise, reducing the possibility of stress appearing during childbirth.

## 5. Conclusions

There are clinical practices that are related to the risk of PTSD, including the type of delivery a woman has, formula-fed newborn at hospital discharge, mother-child separation, her birth plan not being respected, and verbal and psycho-affective obstetric violence. Partner support and initiation of breastfeeding in the first hour were identified as protective factors against PTSD. Practices such as breastfeeding or feeling supported during the birthing process can provide resources for women to empower themselves and cope with a possible risk of postpartum post-traumatic stress disorder, and prevent its onset. Professionals need to be sensitized to this topic since the treatment and care they provide to women can influence the probability of developing PTSD.

## Figures and Tables

**Table 1:** Sociodemographic and current pregnancy characteristics of the sample.

Variable	*n* (%)	Mean (SD)
Maternal age		35.2 (4.25)
Weight before pregnancy (mean)		65.8 (13.24)
Weight after pregnancy (mean)		77.3 (12.96)
Months from last child		5.4 (3.42)
Pre-gestational BMI		24.4 (4.85)
Education level		
Primary school	15 (1.7)	
Secondary school	61 (6.8)	
High school	199 (22.1)	
University	624 (69.4)	
Current working status		
Full-time work	277 (30.8)	
Part-time work	131 (14.6)	
Sick leave	189 (21.0)	
Unpaid leave	103 (11.5)	
Unemployed	199 (22.1)	
Nationality		
Spanish	868 (96.6)	
Other	31 (3.4)	
Family monthly wage		
Less than 1000 euros	46 (5.1)	
Between 1000 and 2000 euros	319 (35.5)	
Between 2000 and 3000 euros	282 (31.4)	
Between 3000 and 4000 euros	78 (8.7)	
Planned pregnancy		
No	64 (7.1)	
Yes	835 (92.9)	
Number of pregnancies		
One	363 (40.4)	
Two	329 (36.6)	
Three	138 (15.4)	
Four	45 (5)	
Five or more	24 (2.7)	
Number of vaginal births		
None	170 (18.9)	
One	402 (44.7)	
Two	283 (31.5)	
Three or more	43 (4.8)	
Place of birth		
Public hospital	736 (81.9)	
Private hospital	152 (16.9)	
Midwife-led hospital	3 (0.3)	
Home	8 (0.9)	
PPQ		9.10 (8.52)
Score < 19	785 (87.3)	
Score ≥ 19	114 (12.7)	

**Table 2:** Bivariate and multivariate analysis between sociodemographic and obstetric characteristics. Obstetric violence and PTSD.

Variable	Obstetric Violence & PTSD Risk			
	Score < 19	Score ≥ 19	OR (95% CI)	aOR (95% CI)
**Maternal age**				
≤35 years	404 (87.3)	59 (12.7)	1 (ref.)	
>35 years	381 (87.4)	55 (12.6)	0.99 (0.67, 1.47)	
**Academic level**				
Primary school	15 (100.0)	0 (0.0)		
Secondary school	53 (88.3)	7 (11.7)	NC	
High school	173 (86.9)	26 (13.1)	NC	
University	544 (87.0)	81 (13.0)	NC	
**Current working status**				
Full-time work	242 (87.4)	35 (12.6)	1 (ref.)	
Part-time work	113 (86.3)	18 (13.7)	1.10 (0.60, 2.02)	
Sick leave	167 (88.4)	22 (11.6)	0.91 (0.52, 1.61)	
Unpaid leave	88 (85.4)	15 (14.6)	1.18 (0.61, 2.26)	
Unemployed	175 (87.9)	24 (12.1)	0.95 (0.55, 1.65)	
**Nationality**				
Spanish	760 (87.6)	108 (12.4)	1 (ref.)	
Other	25 (80.6)	6 (19.4)	1.69 (0.68, 4.21)	
**Family monthly wage**				
Less than 1000 euros	40 (87.0)	6 (13.0)	1 (ref.)	
Between 1000 and 2000 euros	271 (85.0)	48 (15.0)	1.18 (0.48, 2.94)	
Between 2000 and 3000 euros	251 (89.0)	31 (11.0)	0.82 (0.32, 2.10)	
Between 3000 and 4000 euros	154 (88.5)	20 (11.5)	0.87 (0.33, 2.30)	
More than 4000 euros	69 (88.5)	9 (11.5)	0.87 (0.29, 2.62)	
**Planned pregnancy**				
No	56 (87.5)	8 (12.5)	1 (ref.)	
Yes	729 (87.3)	106 (12.7)	1.02 (0.47, 2.20)	
**Maternal antenatal classes**				
No	161 (89.0)	20 (11.0)	1 (ref.)	
Yes (less than 5 classes)	113 (85.6)	19 (14.4)	1.35 (0.69, 2.65)	
Yes (more than 5 classes)	511 (87.2)	75 (12.8)	1.18 (0.70, 2.00)	
**Birth plan**				
No	436 (89.7)	50 (10.3)	1 (ref.)	1 (ref.)
Yes, but not respected	59 (60.8)	38 (39.2)	**5.62 (3.40, 9.28)**	**2.85 (1.56, 5.21)**
Yes, and was respected	290 (91.8)	26 (8.2)	0.78 (0.48, 1.29)	1.49 (0.82, 2.70)
**Twin pregnancy**				
No	769 (87.3)	112 (12.7)	1 (ref.)	
Yes	16 (88.9)	2 (11.1)	0.86 (0.20, 3.78)	
**Live newborn**				
No	3 (50.0)	3 (50.0)	1 (ref.)	
Yes	782 (87.6)	111 (12.4)	**0.14 (0.03, 0.71)**	
**Parity**				
Primiparous			1 (ref.)	
Multiparous			**0.40 (0.25, 0.65)**	
**Induction of labour**				
No	491 (89.8)	56 (10.2)	1 (ref.)	
Yes	294 (83.5)	58 (16.5)	**1.73 (1.17, 2.57)**	
**Natural analgesia**				
No	631 (86.7)	97 (13.3)	1 (ref.)	
Yes	154 (90.1)	17 (9.9)	0.72 (0.42, 1.24)	
**Regional analgesia**				
No	232 (91.0)	23 (9.0)	1 (ref.)	
Yes	553 (85.9)	91 (14.1)	**1.66 (1.03, 2.69)**	
**General anaesthesia**				
No	763 (87.8)	106 (12.2)	1 (ref.)	
Yes	22 (73.3)	8 (26.7)	**2.62 (1.14, 6.03)**	
**Type of birth**				
Normal vaginal delivery	500 (92.8)	39 (7.2)	1 (ref.)	1 (ref.)
Instrumental	150 (88.2)	20 (11.8)	1.39 (0.92, 2.09)	1.08 (0.56, 2.11)
Elective C/S	48 (80.0)	12 (20.0)	1.08 (0.56, 2.08)	**2.53 (1.02, 2.26)**
Emergency C/S	87 (66.9)	43 (33.1)	**2.09 (1.35, 3.23)**	**3.58 (1.83, 6.99)**
**Episiotomy**				
No	567 (86.2)	91 (13.8)	1 (ref.)	
Yes	218 (90.5)	23 (9.5)	0.66 (0.41, 1.07)	
**Perineal tear**				
No	453 (84.5)	83 (15.5)	1 (ref.)	
Mild	306 (92.2)	26 (7.8)	**0.46 (0.29, 0.74)**	
Severe	26 (83.9)	5 (16.1)	1.05 (0.39, 2.81)	
**Skin-to-skin**				
No	135 (71.4)	54 (28.6)	1 (ref.)	
Yes	650 (91.5)	60 (8.5)	**0.23 (0.15, 0.35)**	
**Breastfeeding 1 h after childbirth**				
No	171 (78.1)	48 (21.9)	1 (ref.)	1 (ref.)
Yes	614 (90.3)	66 (9.7)	**0.38 (0.25, 0.58)**	**0.48 (0.26, 0.87)**
**Admission of the new born to care unit**				
No	699 (89.8)	79 (10.2)	1 (ref.)	1 (ref.)
Intermediate care	38 (64.4)	21 (35.6)	**4.89 (2.73, 8.75)**	**4.95 (2.36, 10.36)**
NICU	48 (77.4)	14 (22.6)	**2.58 (1.36, 4.89)**	**2.25 (1.02, 4.97)**
**Place of birth**				
Public hospital	646 (87.8)	90 (12.2)	1 (ref.)	
Private hospital	128 (84.2)	24 (15.8)	1.35 (0.83, 2.19)	
Midwife-led hospital	3 (100.0)	0 (0.0)	0.00 (0.00, 0.00)	
Home	8 (100.0)	0 (0.0)	0.00 (0.00, 0.00)	
**Hospital length of stay**				
1 day	66 (94.3)	4 (5.7)	1 (ref.)	
2 day	412 (93.2)	30 (6.8)	1.20 (0.41, 3.52)	
3 day	203 (83.9)	39 (16.1)	**3.17 (1.09, 9.20)**	
4 days or more	104 (71.7)	41 (28.3)	**6.51 (2.23, 19.00)**	
**Partner support during childbirth**				
None	8 (44.4)	10 (55.6)	1 (ref.)	1 (ref.)
Little	19 (54.3)	16 (45.7)	0.67 (0.22, 2.11)	0.74 (0.19, 2.86)
Something	47 (83.9)	9 (16.1)	**0.15 (0.05, 0.49)**	**0.16 (0.04, 0.63)**
Quite	198 (88.0)	27 (12.0)	**0.11 (0.04, 0.30)**	**0.17 (0.05, 0.57)**
A lot	513 (90.8)	52 (9.2)	**0.08 (0.03, 0.21)**	**0.17 (0.06, 0.53)**
**Infant feeding on discharge**				
Maternal	362 (90.0)	70 (10.0)	1 (ref.)	1 (ref.)
Mixed	126 (78.3)	35 (21.7)	**2.51 (1.60, 3.91)**	1.42 (0.81, 2.49)
Artificial	27 (75.0)	9 (25.0)	**3.01 (1.36, 6.66)**	**3.57 (1.32, 9.69)**
**Postpartum surgical intervention**				
No	759 (88.1)	103 (11.9)	1 (ref.)	
Yes	26 (70.3)	11 (29.7)	**3.12 (1.50, 6.50)**	
**Maternal ITU admission**				
No	785 (87.3)	114 (12.7)	NC	
Yes	0 (0.0)	0 (0.0)		
**Hospital readmission**				
No	764 (87.6)	108 (12.4)	1 (ref.)	
Yes	21 (77.8)	6 (22.2)	2.02 (0.80, 5.12)	
**Verbal violence**				
No	632 (93.9)	41 (6.1)	1 (ref.)	1 (ref.)
Yes	153 (67.7)	73 (32.3)	**7.36 (4.83, 11.21)**	**5.07 (2.98, 8.63)**
**Physical violence**				
No	381 (93.2)	28 (6.8)	1 (ref.)	
Yes	404 (82.4)	86 (17.6)	**2.90 (1.85, 5.54)**	
**Psych-affective violence**				
No	544 (95.6)	25 (4.4)	1 (ref.)	1 (ref.)
Yes	241 (83.0)	89 (27.0)	**8.04 (5.03, 12.84)**	**2.61 (1.45, 4.67)**
**Violence (dichotomous)**				
No	283 (96.6)	10 (3.4)	1 (ref.)	
Yes	502 (82.8)	104 (17.2)	**5.86 (3.02, 11.40)**	

Bold: Statistically significant differences. OR: Odds ratio. aOR: Odds ratio adjusted.

## Data Availability

The datasets generated and/or analyzed during the current study are available from the corresponding author on reasonable request.
